# Book Reviews

**Published:** 1832-10

**Authors:** 


### CRITICAL ANALYSES.

Quæ laudanda forent, et quæ culpanda, vicissim,
Illa prius, cretâ; mox hæc, carbone, *notamus.*—PERSIUS.

*Practical Observations in Midwifery; with a Selection of Cases.* Part II. By JOHN RAMSBOTHAM, M.D., late Lecturer on Midwifery at the London Hospital, &c.—8vo. pp. 507. S. Highley, London.

SEVERAL years have elapsed since the publication of the first part of Dr. RAMSBOTHAM's "Practical Observations in Midwifery," and he now, fortunately for the obstetrical student, has completed his very useful undertaking. Both in this country and on the continent, the former part of the work was very favorably received by the profession; and one most competent judge of its merit, Dr. DEWEES, of Philadelphia, considered it of sufficient practical importance to induce him to republish it in America; and in the preface to his edition, he mentions it as "one of the best practical works (so far as it goes) extant." In this opinion we fully concur, and, after a careful examination of the second part of the work, (of which we have now to give an account,) we feel justified in extending to it an equally favorable verdict.

The present volume contains the history of one hundred and thirty midwifery cases of various kinds and different degrees of interest and difficulty, all of which, with very few exceptions, have occurred in Dr. R.'s practice. Each division of the cases is preceded by brief practical remarks, in which those points are particularly commented upon which are most likely to perplex or deceive the obstetric student or young practitioner in midwifery, and in which also the best mode of treatment is described, where circumstances of danger or difficulty exist.

The first subject to which Dr. R. directs our attention is the *breech presentation*, which, on a general average and rough calculation, he thinks may be estimated "at about one in thirty cases." The relative frequency of such cases is not, perhaps, a matter of great importance; but we confess we are surprised to find that the very extensive experience of the author has led to such a conclusion. We doubt whether the *general* average would produce more than one case of breech presentation in *sixty*. We pass over the brief outline which is given of the natural progress of a common breech case in which there is neither deficiency of pelvic space nor defect of uterine action. The directions that are given for the management of such cases, when the trunk has passed as far as the shoulders, are extremely good, and merit the particular attention of the novice in the practice of midwifery; and, as experience is by no means synonymous with knowledge, some well-practised men may look to them with benefit. We make this remark in consequence of having more than once known the life of the child sacrificed, if not to the ignorance, to the still more culpable carelessness of the accoucheur, in such cases. As soon as the breech and legs are excluded, the umbilical cord becomes exposed, and the state of the fœtal circulation may now be determined.

"If the cord is at this moment drawn tightly upward, partly from retraction and partly from pressure, the fœtal circulation is in great danger of permanent interruption. To prevent the mischievous consequences likely thence to ensue, a fold or two of the funis may be cautiously brought down. The true condition of the fœtal circulation will thus be ascertained, and by that condition must the future proceedings be in a great measure regulated.

"If umbilical pulsation is found to be going on vigorously and uniformly, the further expulsion of the trunk may be safely intrusted to uterine action; the more especially, if that action is continuing regular and effective. But if that pulsation begins to flag and to intermit, whereby its cessation is threatened; or if the returns of uterine action become more distant or less powerful; the delivery of the other parts of the child must not be delayed. Yet the extractive efforts should be applied in so gentle and gradual a manner as to inflict no additional pain upon the mother; and always under the impression that the child may be born alive. When extractive assistance becomes thus necessary, the occasional application of a hand upon the uterine tumor will enable the accoucheur to judge whether the degree of uterine contraction is proportionate to that of fœtal extraction. If the child after birth does not speedily shew a disposition to breathe, the most effective measures should be promptly had recourse to for its resuscitation; for a restoration from that state of suspension under which animal life is probably at that instant languishing. Of these, immersion in warm water, with inflation of the lungs, holds the first rank. In every case of breech presentation, therefore, it becomes a matter of prudent foresight to provide such means in immediate readiness for use, as may be wanted under the above emergency.

"But if, on the other hand, upon the free exposure of the funis, no pulsation can be detected, the child is, in all probability, already bereft of life; yet, of that fact, there is at present no direct evidence; for the umbilical circulation may be only temporarily intercepted by pressure, and the child may be at the moment under a state of suspended animation. Between these two states, there is a most essential difference; yet I cannot mention any characteristic mark by which one can be distinguished from the other. We must therefore be guided by probabilities; and if, after some pause, no further pulsation can be discovered in the funis, I should be disposed to think that life was extinct in the child. Under that impression, I should not be anxious to hurry the extraction of the shoulders and head. Yet, if the slightest umbilical pulsation is perceptible, if there is an obvious feeling of motion, or if the mother expresses a decided conviction that her infant was living but a short time before, immediate extraction ought not to be delayed.

"In some cases, in which the breech, legs, and part of the trunk are in the world, while the shoulders and head are remaining behind, I have observed a distinct heaving of the chest and belly under my hand, indicative of a futile attempt on the part of the child to inspire. I have also remarked that, when this occurrence has taken place, the child has been born, either under a state of suspended animation from which it has been with difficulty recovered, or of actual deprivation of life. This attempt at inspiration is induced, I presume, by the premature exposure of the body to atmospheric air; and when it does occur, it ought to prove an additional stimulus to activity in the extrication of those parts which are still unborn. Whether the child in that act inhales some gaseous vapour injurious to the respiratory organs, or draws into the air-passages some portion of the vaginal fluids, is a matter of little importance; the child's life is placed thereby in the most imminent hazard, from which it can only be rescued by the speedy release of the head." (P. 12.)

Cases sometimes occur in which, either from deficiency of pelvic space or from defect of uterine action, or perhaps from a combination of both these causes, the breech becomes impacted in the pelvis, and demands extractive assistance for its release. To such cases Dr. R. especially adverts.

"When the breech remains stationary in the pelvis for a length of time, under a due continuance of uterine action, its detention must arise either from relative mal-position, or from disproportion between the breech and the passage. Under this detention, it is no uncommon occurrence to meet with considerable tumefaction in the nates and parts of generation of both sexes, especially in the scrotum of a boy. The swelling arises from obstruction to the return of the circulating fluids, in consequence of continued pressure upon the parts above; and sometimes proceeds to that extent in a boy, as to make the scrotum discoloured, and so much enlarged as to appear externally, even when the breech is not near the external parts. At the commencement of this swelling the child is undoubtedly alive, yet the embers of life may possibly be extinguished before the child is expelled; at any rate, the life of the child is in a state of danger.

"If the breech has remained unmoved so long, and with such symptoms, in the situation above described, as to induce a well-grounded suspicion that it will not pass without some extractive assistance, the cause of its detention must be carefully explored. In case there is not much impaction, with the breech well down in the pelvis, the assistance of a finger may be sufficient to overcome the obstruction, and to induce such a change in the situation of parts as may terminate in expulsion or extraction: for, when once a slight advantage is obtained, the difficulty is presently surmounted. A fore-finger of that hand which appears the better fitted for the purpose, may be carried in a hook-like form over one of the groins or thighs of the child, by which an extractive purchase may be gained, sufficient to overcome a slight degree of arrest. But if the impaction proves considerable; if the outlet of the pelvis is deficient in space; or if the breech is situated so high as not to be within the full reach and power of the finger; some other means must be resorted to, affording a superior degree of purchase. In such a case a blunt hook may be carefully insinuated over one of the groins of the child, with which traction downward must be cautiously and gradually made." (P. 22.)

If, in a breech presentation, the pelvis is deformed at the upper strait, the difficulty of delivery will be in proportion to the degree of deformity there existing. Dr. R. states it as his opinion, that if there is a less space than three inches in the conjugate diameter, the breech of a full-grown child will either not pass at all, or will be propelled through with great difficulty. It will constantly happen in practice, when there is but a slight degree of deformity, that hour after hour is allowed to pass away in anxious expectation of the descent of the breech, until the expulsive powers are much diminished in strength, or become altogether exhausted. In such a dilemma, a recourse to extractive assistance becomes eventually necessary, and of course more absolutely so, and at a far more early period, when the deformity is considerable. Admitting that, as a general principle, the application of artificial assistance on slight grounds is justly censurable, the author very properly observes, that the withholding of it until exhaustion is positively approaching is by far more blameable.

"Not only is a greater degree of extractive purchase then required, but there is also increased danger of the infliction of injury upon the mother's parts. The mode of proceeding is similar in each case, but in one the exertion of more power is required. The necessity for artificial assistance being satisfactorily established, an accurate estimate of the pelvic deficiency, as well as of the state of the soft parts, must be obtained by a suitable examination. For this purpose, it may become necessary to introduce two or more fingers, and even the greater part of the hand, within the vagina. If the degree of pelvic deformity is found to be so considerable as to preclude the extraction of the breech without the application of great force, as soon as the state of the soft parts will permit, the operation must be commenced, and prosecuted with due perseverance to the extraction of the whole of the child; and, in this act, the assistance which will be derived from expulsive uterine action will be found most essentially useful. This fact alone shews the impolicy of long delay." (P. 25.)

The mode of expediting the delivery in such instances is accurately described. Several cases of difficult breech cases are detailed, and we select the following as the most instructive.

"*A very difficult Breech Case, from Deformity of the Pelvis.*

"About noon, Wednesday, November 9th, 1814, I was called to the assistance of Mrs. M., in Finsbury district, in labour of her first child, a woman of low stature, and above forty years of age. Her labour-pains had commenced on the evening of the Monday preceding, and soon afterwards her medical attendant was summoned, who had been with her the greater part of the intermediate time. On examination, I immediately detected the breech lying above the brim of the pelvis; which, as far as I could judge from the nicest measurement I could make, did not possess a space equal to two inches from pubis to sacrum. As the os uteri was but little dilated, and the woman's strength not much impaired, I merely recommended for the present an occasional enema. I visited this patient again at six in the evening: at this hour, the os uteri was become a little more open, as well as more flaccid; the labour-pains were more forcing; but no descent of the breech could take place for want of space. Seeing no possibility of improvement, I now determined upon immediate delivery, which I foresaw would be a task of no little difficulty. Having introduced my left hand within the vagina, I passed its fore-finger over one of the groins of the child, and upon it I insinuated a blunt hook, which gave me an excellent purchase. After exerting a good deal of force, I managed to get down the leg of that side. I then carried the blunt hook over the other groin, and got down the other limb in a similar manner. The possession of the legs enabled me to extract the trunk as far as the axillæ. The arms were at this time drawn up on each side of the head; with some difficulty, I carried the blunt hook over one of these, which enabled me to bring it down. I proceeded in a similar manner with the other arm; but the most difficult part of the delivery I had yet to encounter in the extraction of the head; for it was impossible that the head could pass entire through such a pelvis, and it seemed to me to be no easy matter to perforate it. After a careful inquiry into its exact position, I brought the occiput close behind the pubes; then passing the two fore-fingers of my left hand against the under part of the occipital bone, with the perforator I made a large and free opening through the skull into the brain. Within this opening I inserted the blunt hook, and getting thereby a very firm purchase upon the base of the skull, by the continuance of the extractile power it afforded, under the exertion of which the contents of the cranium were largely expelled, I succeeded in extracting the head. The placenta followed immediately. The next day the woman had procured refreshing sleep during the night; had passed urine; and, indeed, seemed as comfortable as if she had undergone no unusual inconvenience. She ultimately recovered as well as after the most natural labour, and is still living." (P. 32.)

Two instructive cases are briefly detailed, in which much difficulty was produced by improper attempts at extraction in breech presentations.

The remarks made by Dr. R. upon the subject of shoulder presentations are very judicious. Having satisfactorily ascertained that the shoulder is the presenting part, one general rule of practice is applicable to every case of this kind, if the period of gestation is nearly completed. The child must be turned and brought down by the feet. We are cautioned not to suppose that natural expulsion is never effected when the child is thus situated; "but such expulsion is so seldom accomplished by uterine agency alone, so great a degree of hazard is also incurred by awaiting that rare event, that it has now become the established practice to turn the child by art, as a preferable and a far less dangerous mode of proceeding." It may sometimes be impossible to introduce the hand at all, from the powerful contraction of the uterine parietes upon the body of the child. In such untoward instances, the destruction of the child may be absolutely necessary, that the more valuable life of the mother may be saved; but so desperate an alternative should never be had recourse to without a consultation, and we find from the cases he details that Dr. R. himself, although he does not, in his introductory observations upon the subject, particularly enforce this prudent and necessary precaution, usually takes another opinion before he performs the very disagreeable, but yet sometimes necessary, duty of destroying the infant. In those difficult cases in which the shoulder has become impacted in the pelvis, with the hand and arm external, and in which a previous attempt to turn has not proved successful, some practitioners have recommended the removal of the arm at the shoulder. We perfectly agree with the remarks that Dr. R. offers upon this point: he says that the practice adverted to is proposed under the delusive idea of diminishing the general bulk of the presenting part, and of permitting a more ready entrance to the hand for the purpose of turning; but he contends that it cannot satisfactorily effect either of these objects. In his opinion, indeed, such an act can only increase the previous difficulties.

"The operation must be performed either by means of some cutting instrument, or the arm must be forcibly twisted off by the exertion of violence. By either mode, a larger portion of the child must necessarily be drawn down, and more firmly wedged within the pelvis. But even after the removal of the arm, no advantage is gained; for the shoulder and chest remain in the same state, or perhaps in a more impacted state, with all the previous obstacles to the introduction of the hand. Besides, such a degree of confusion is thereby produced within the vagina, as to make it almost impossible to discriminate the parts of the child from those of the mother. I therefore consider the dismemberment of a descended arm to be rather detrimental than beneficial." (P. 61.)

In the opinions he gives upon this subject, Dr. Ramsbotham is supported by the authority of Denman.* Dr. R. has long discarded from his practice the use of opium, which is not unfrequently given for the purpose of diminishing uterine power, and thus removing some part of that contraction which resists the admission of

* "It is in no case necessary, or in anywise serviceable, to separate the arm of the child previous to the introduction of the hand of the operator."—*Waller's edition of Denman's Midwifery*, p. 352.

the hand. He believes that these expectations will generally be disappointed. May not the abstraction of blood be sometimes useful in diminishing the more powerful contraction of the uterus upon the body of the child? Several cases of shoulder presentation are related.

In the following instance it is more than probable that, if the endeavours to turn had been persisted in, the life of the mother would have been lost.

"*Decapitation under a Shoulder Presentation.* At four P.M., Wednesday, August 28th, 1822, a note was sent to me from one of the midwives of the charity, requesting my assistance to a poor woman in Wheeler street, Spitalfields, and stating, 'that she had been in labour of her first child twenty-four hours, that the waters had been discharged one hour, and that the hand was presenting.' I found an arm low down in the pelvis, the shoulder completely blocking up its brim, and the uterus firmly contracted upon the child. I attempted to introduce my hand, and to push up the shoulder; but I met with such a degree of resistance from uterine contraction in that attempt, that I did not think it prudent to persist in my endeavours to turn. After the lapse of a short time, I decided upon decapitating the child: with some difficulty I got my finger over the neck, upon which I passed a blunt hook, and by its side the decapitator; then, withdrawing the blunt hook, I brought the instrument without much difficulty through the neck of the child. The shoulders, trunk, and lower extremities, were now extracted with ease by the descended arm; the head was afterwards brought away by means of a blunt hook inserted into the mouth, and the placenta immediately followed. The woman did well.

"It appeared to me in this case, that if I had resolutely persisted in my endeavours to turn, I must either have lacerated the uterus, or have inflicted other irreparable mischief upon the soft parts of the mother. In cases of strong tonic contraction of the uterus, the child must almost necessarily be produced into the world dead; the act of decapitation or of exvisceration, therefore, will lose much of its apparent violence; yet either can never be justified, except upon the principle that, through its means, less injury may probably be inflicted upon the mother, than by the usual mode of turning." (P. 100.)

The comments upon the general subject of uterine hemorrhage, and the many very interesting cases that are related, are especially valuable, and cannot fail to interest the student.

*Puerperal Convulsions.* Dr. Ramsbotham has not been able to obtain satisfactory evidence, at least from his own observation, that an attack of this formidable malady could be induced by domestic affliction or mental anxiety, but he has repeatedly remarked, among the numerous patients of the Royal Maternity Charity, as well as among others to which he has been accidentally called, that several cases have occurred soon after each other. Whether this fact ought to be attributed to mere chance, as to the agency of some general or influence upon the female system, he leaves to others to determine; but he suspects it may be attributed to the latter principle. He observes also, that he has witnessed the occurrence of several cases during warm weather; at a time when the clouds have been charged with elective fluid; when atmospheric appearances have threatened a thunder storm; and when perhaps they have ended in one. The whole train of symptoms of puerperal convulsions evinces great derangement in the functions of the brain and nervous system; yet, says Dr. R., correspondent marks of organic mischief within the brain are seldom met with. The different anatomical inquiries at which he has been present have not disclosed such regular appearances as to sanction the uniform deduction that the brain was the principal seat of the disease. He suspects that, in many instances, that important organ is no otherwise implicated than through the medium of sympathetic irritation. We have more than once had reason to agree in opinion with our author upon this point. Active bleeding and purgatives are the remedies principally to be depended upon in puerperal convulsions.

"If it should be found that the practice above recommended produces, within a short time, no palliation of the symptoms, relief must be sought, without much delay, in delivery; especially if the process of labour has obviously commenced. It is presumed, that the convulsive paroxysms are some way or other connected with the state of pregnancy; if, therefore, those means fail, the labour must be terminated by art, whatever may prove to be the result. When the os uteri is so far dilated as to admit the easy introduction of the hand, or when it is in a state to permit a ready extension thereby, the hand must be passed into the uterus, the feet engrasped and brought down, after which the labour may be completed at pleasure. But before recourse is had to turning the child, there ought to be a satisfactory conviction in the mind that the state of parts will allow the act to be accomplished easily, readily, and without the infliction of injury. Should the attempt be made at all hazards, without reference to vaginal or uterine relaxation, it might either be entirely foiled, or effected under such violence as greatly to enhance the risk of danger. In this, as in every other case in which a recourse to artificial delivery becomes absolutely indispensable, the operation must be considered in no other light, than as entirely subservient to the present safety and future welfare of the patient." (P. 259.)

Upon a general average of cases, Dr. R. thinks it will be found that convulsions after delivery are more intractable, and prove more frequently fatal, than when they occur previous to or during labour. He has remarked that when they come on under either of the latter states, and *continue after delivery*, whether it may have been effected naturally, or hastened by art, they generally prove destructive to the patient; "but that, if they be checked by delivery, they seldom return afterwards; a quiet sleep presently succeeds, which is usually the first and most favourable harbinger of subsequent recovery. After free evacuations, the injection of an enema, composed of a proper quantity of an opiate, with assafœtida or turpentine, has in some cases seemed beneficial. Dr. R. details several cases of puerperal convulsions occurring both before and after delivery: some of them terminated fatally, and others were relieved by delivery or bleeding. We give one of the successful cases.

"*Convulsions during Labour, relieved by Delivery.* About one in the morning of Thursday, September 10th, 1818, I was called to the assistance of a lady in Fenchurch street, who had been a few hours before seized with convulsions during the progress of a natural labour under a first child. The patient was a stout, corpulent, well-looking, young woman, about twenty years of age. The pains of labour came on the preceding forenoon about ten, and went on regularly till about four in the afternoon, when the membranes gave way, and the attending gentleman was sent for. After this time, the labour advanced slowly but progressively till about ten in the evening, when the woman was seized with a convulsion fit without any previous notice. A vein was immediately opened in the arm, and about fourteen or sixteen ounces of blood were taken away. Between this hour and my arrival at the bedside, she had two more fits. I found her completely senseless, snoring like a patient under apoplexy; yet it was evident that she had occasional labour-pains, from an alteration in her manner, and appearance during their presence. While my friend and I were conversing upon the case, another violent paroxysm took place. Venesection was immediately repeated, and twenty ounces of blood were again abstracted. Upon making a vaginal examination, I found the head occupying the cavity of the pelvis, and sufficiently within the scope of the forceps; and thinking that immediate delivery would prove the most likely means of averting the threatened consequences, I set about the operation by means of that instrument. After its satisfactory application, I had to encounter considerable difficulty in the extraction of the head, arising from the struggles of the patient, and her incessant change of posture. When the head was brought down so low as to press upon and to extend the external parts, another paroxysm occurred more violent than the preceding ones; after which the child was slowly extracted, and the placenta soon followed.

"During the delivery, this woman appeared to be insensible both to my efforts and to uterine contraction, and continued to snore as before; but after delivery she became more composed, the snoring ceased, and she seemed to be comfortably asleep; so that I left her with a strong impression of her future safety. At my visit early the next morning, I found her very much recovered, indeed making little complaint: as general appearances were so favorable, I took my leave, intrusting her future management to her previous attendant.

"The child was born alive, but in a very weakly state. Upon immersion in warm water, the infant rallied, and cried stoutly; notwithstanding, after a short time, this child began to droop, and died about twelve hours after birth." (P. 308.)

Our limits oblige us to pass over the sections on *Labour with two or more Children*, and on *Abortion*, although upon both these subjects the remarks made by the author, and the cases he gives, are of much interest.

The following "*Anomalous Case, assuming many appearances of Pregnancy*," shews how difficult it sometimes is, even for the most experienced, to form a correct diagnosis.

"In the evening of Tuesday, December 5th, 1826, I visited a lady in consultation with a most respectable professional gentleman at a short distance from town, the mother of a large family, who had been seriously indisposed for some weeks, and was supposed to be in the sixth month of pregnancy. She was complaining of considerable pain in her back; of an intolerable itching irritation throughout the whole surface of the skin, and of tenderness in the abdomen. She had a small quick pulse; a coated tongue, and a harassed countenance. But the most prominent symptom was an excessive enlargement of the uterine tumor, with a painful extension of the whole abdominal parietes, far exceeding the common size of the belly at this period of pregnancy; equal indeed to that at the end of gestation, and offering an equal degree of resistance under the hand. This unusual enlargement had chiefly taken place within the last fortnight, and did not possess any obvious fluctuation. A vaginal examination added little information to that already obtained by external inquiry. The os uteri was slightly open, thin, and relaxed; the cervix was extended, and scarcely perceptible. Under the impression of pregnancy, this abdominal extension could only be referred to a morbid deposition of liquor amnii; a surmise verified in the result; yet present interference did not appear to all parties desirable; a temporizing plan was, therefore, for the present recommended. At a second visit the following day, the lady had passed a restless night, and had been much annoyed by the irritation on the skin. She continued in nearly a similar state to the afternoon of Friday the 8th, when parturient pains commenced, the os uteri opened, the membranes protruded, but no part of the child could be felt by her medical friend. After some time the bag of membranes spontaneously gave way, and a very extensive rush of liquor amnii instantly followed. The uterus presently contracted, and in due time expelled a child and placenta apparently between the fifth and sixth months. After this event, the common occurrences subsequent to labour ensued; the abdominal extension subsided, and the late unpleasant symptoms gradually disappeared. But on Tuesday, December 12th, this lady was attacked with febrile symptoms, accompanied by pain in the belly; but these were soon relieved by leeching and purging: she was so much recovered on Saturday the 16th, being comparatively free from complaint, that I took my leave.

"On Wednesday, December 12th, 1827, little more than a year from the preceding date, I was again called in consultation with the same parties. This lady was now suffering under general febrile symptoms, attended with considerable irritability of the stomach, which rejected almost every article of medicine and diet; and with, as in the former instance, excessive irritation over the whole surface of the skin. She had a dejected countenance; a small rapid pulse; a clammy mouth, with a white tongue; and was supposed to be somewhat more than three months advanced in pregnancy; yet there was a size of belly equal to that of most women near the end of gestation. The uterine tumor was distinctly felt under the hand large, firm, and resistent, and was extremely painful on pressure throughout its whole extent; but there was one point towards the right ilium, where its sensibility was greater than in other parts; the slightest touch was there bitterly complained of. More or less of a constant draining discharge had escaped from the vagina for the preceding five or six weeks, which had been sometimes purely sanguineous, but at others had been of a serous description, with the occasional appearance of small coagula upon the napkins; in greater quantity during the night-time, but devoid of unpleasant smell. The uterine growth had increased gradually to its present size; and although, under a suspicion of pregnancy, abortion had been expected, no indication of uterine action had yet appeared. A small quantity of blood had been taken away at the commencement of this illness without advantage; and the present state of this lady forbad a repetition of that operation. Looking at the similarity of the symptoms to those above described in her last pregnancy, I was induced to attribute the abdominal enlargement to the same cause; viz. to a morbid deposition of the liquor amnii; but the sequel will shew that it was the result of an uterine disease of a most singular character. The treatment was, for the present, merely directed to the relief of the more urgent symptoms by aperients and opiates.

"During the interval between her recovery from the preceding confinement and the first week in August, this lady had enjoyed her usual state of health; no vestiges of indisposition remained, except in the appearance of the countenance, which had not regained its usual aspect; and to the time just mentioned she had menstruated regularly and correctly. The catamenia then became interrupted, and afterwards a gradual yet very unusual increase of size followed. Such symptoms naturally induced a suspicion of pregnancy in her own mind, in that of her husband and friends, as well as in that of her medical attendant.

"On Friday morning, December 14th, the husband called upon me to say, that his wife was getting worse, and that he was desirous of a consultation with a celebrated accoucheur now no more. Accordingly an appointment was made at four P.M. that day. Each made a vaginal examination; but the presence of pregnancy could not be satisfactorily detected. The cervix uteri was elongated and thickened; the os uteri was soft, flaccid, and a little open, so as to admit the passage of the finger about half an inch within the cervix; yet nothing like membrane was perceptible, and the general mass of the viscus was obviously much enlarged. Now, although these appearances did not indicate a state of regular gestation, they were in many respects so similar to those under her last pregnancy, that we were strongly inclined to believe the lady again in that state. Under that impression, the treatment was merely palliative and temporizing.

"I visited this lady again on Monday the 17th, when I found the abdominal extension evidently and rapidly upon the increase, and its external surface equally painful to the touch; in other respects there was little variation in the general symptoms. On Wednesday the 19th she was much worse, and it seemed to me sufficiently apparent that exhaustion must presently ensue, unless some effectual means could be devised to check the progress of these alarming symptoms. Another consultation with the same parties was had at four P.M. Thursday the 20th. It was then remarked, that the lady had lost much ground within the last few days, and that the abdominal size was rapidly increasing. Under the impression of pregnancy, a catheter was introduced high within the uterus, with the intention of discharging the liquor amnii, but none escaped; the instrument passed without obstruction, and was easily moved about, as if *in vacuo.*

"At ten A.M. Friday the 21st, I was called in a hurry in consequence of the occasional recurrence of pains which were presumed to be indicative of uterine action. At my arrival they had subsided, but symptoms of a most alarming kind had ensued. The pulse was quick and tremulous; respiration was frequent and laboured; the countenance was sunk, with appearances of rapid exhaustion; yet the lady was at this time perfectly sensible, and there had been no increase of uterine discharge. The rapid approach of dissolution was too obvious, which took place a little before noon.

"The body was inspected, under a state of considerable decomposition, on Saturday evening, December 22d. The abdomen was tumid and soft under the hand, having lost its former firmness. On the division of its parietes, a quantity of very offensive gas escaped. The uterus had an oviform shape, and was in size equal to that at the sixth month of pregnancy; it was flabby under the hand, and had a red appearance on its external surface, yet not indicative of inflammatory action. The stomach and intestines were distended by gas; the other viscera had a healthy aspect. Upon removing the uterus out of the pelvis, the os uteri was open and flaccid, and within it was seen a tinge of dark redness, which was afterwards found to pervade the whole of its inner surface. Upon dividing the uterine structure, its parietes where thickened and enlarged, as under pregnancy, but no appearance of a fœtus could be detected. The cavity merely contained a fibrous mass, about the size of a large egg, loosely attached to the posterior surface, and entangling within its substance a number of small coagula. This mass was surrounded by a quantity of bloody purulent fluid, in which were floating other small coagula. The whole of the internal surface of the viscus was in a state of disease, which on a close inspection put on the appearance of granulating eminences; in some points, especially near the cervix uteri, its structure was destroyed by the ulcerative process, almost to the peritoneal covering. The uterine cavity would have contained a body equal in size to the infantile head at full time. The ovaria, the fallopian tubes, and the vagina, had a healthy appearance.

"This case exhibits a singular instance of uterine development and increase, in a disease of the uterine membrane of an ulcerative kind, which shewed many of the external appearances of pregnancy. The enlargement and growth of the uterine parietes were apparently the result of a natural effort to counteract the baneful tendency of this dangerous affection; and the sanguineous and sero-sanguineous discharges were the necessary consequences. I think it extremely probable, that the large and comparatively sudden deposition of the liquor amnii in the preceding pregnancy was connected with the existence of this disease in its incipient state, and that the uterine membrane never attained a state of perfect health after the expulsion of its last contents. The principal difference between the enlargement of the uterus in the present case, and that under pregnancy, seems to be, that the former is accompanied with pain, the latter is free from that inconvenience; yet neither is similar to that extension which is the consequence of copious amnial deposition."

In the subsequent pages Dr. R. relates several curious cases of retroverted uterus, and of polypus uteri, and also three instances of recovery with rupture of the uterus. We must conclude our notice of Dr. R.'s very valuable work, which we cannot too strongly recommend to the profession, with the following "*awful case of sudden death in the last month of pregnancy.*"

"I was engaged to attend a young married lady in West Smithfield, who expected to be confined of her first child, the first or second week in January 1812. She went into the immediate neighbourhood of her own house, to join a party of friends on the evening of New-year's day, being at that time apparently in perfect health; and after she had been among them a few hours, in full enjoyment of the hilarity of the evening, she suddenly complained of being very ill. With great difficulty she was got up stairs, and was seated in an easy nursing chair; presently she fell lifeless on the floor! The people about her supposed her to be in a fainting-fit: a neighbouring medical man was called, who, in the first instance, thought the lady in a state of syncope, but presently pronounced her to be dead! Some time was lost in the confusion, which prevailed in the house; but, by and bye, a messenger was despatched for me. I arrived at the house a little after midnight; at that time, the lady had been lifeless at least an hour: notwithstanding, I would gladly have removed the child by the cæsarean section, but her friends would not consent to that operation.

"Leave was obtained to inspect the body the next day, yet only on condition that a near relative should be present. On dividing the abdominal parietes, the gravid uterus presented itself to view, but very different in its aspect from that which is generally met with. The whole of the fore part of the fundus, and some portion of the back part of the uterus, was completely black; not unlike that appearance upon the skin of a delicate woman, after the infliction of a severe blow. The fallopian tubes were turgid and black; the ovaries were of a natural size, but they had a striated or speckled appearance, somewhat like mottled soap. Upon making an incision into the peritoneal coat of the uterus at its back part, where the black or suffused appearance was the most obvious, fluid blood freely followed the knife. The placenta was attached at the fore part of the body of the uterus throughout its entire extent, and the child was presenting naturally; the internal uterine surface seemed healthy. The stomach, the intestinal canal, and the other abdominal viscera, had the usual healthy appearance. The heart and the large blood-vessels were healthy and sound; within the pericardium was contained a small quantity of serous fluid; the right lung was a little diseased, with trifling adhesions to the pleura costalis; the left lung was healthy. The head was not allowed to be examined.

"The above appearances led me to suspect that some large vessel had given way within the uterine structure, the contents of which had been effused into the cellular tissue under the peritoneal coat; producing a state in the gravid uterus similar to that of the brain under effusion beneath its meninges." (P. 505.)

---

*Observations on the General Principles and on the Particular Nature and Treatment of various Species of Inflammation.* By J. H. James, Surgeon to the Devon and Exeter Hospital, and Consulting Surgeon to the Exeter Dispensary.—8vo. pp. 565. Renshaw and Rush, London.

To whatever branch of the sciences of medicine or surgery the practitioner may devote his attention, an intimate knowledge of the causes, effects, terminations, and modes of treating the various species of inflammation, to which the different structures of the body are exposed, must be considered as the stepping-stone by which he is to rise to a satisfactory acquaintance with the nature of the great majority of both local and general diseases. It is to be lamented that much doubt, and even mystery, still hang over many physiological questions which relate to that morbid condition to which we apply the abstract term *inflammation.* The change which exists in the action or condition of the blood-vessels of an inflamed part is not yet so satisfactorily established as to leave no further room for difference of opinion, nor can we venture to assume that the part which the nervous system plays in the production or modification of inflammatory action has been hitherto clearly established: but if, as far as regards the *essence* of inflammation, we still feel ourselves involved in some obscurity, we have, on the other hand, an abundance of well-ascertained practical facts concerning its different varieties, the progressive changes which an inflamed part undergoes, and the best modes of treating inflammation, the study of which must amply repay the student, and the accurate knowledge of which he must obtain before he can, either conscientiously or efficiently, undertake the duties of a medical or surgical practitioner. Now, for this essentially necessary information, we cannot better consult the interest of the student than by advising him to take the present volume as his guide. The arrangement of the subject adopted by Mr. JAMES appears to us unexceptionable: his style of writing is concise and perspicuous, and his purpose is to convey practical information, rather than to entangle himself or his readers in speculative doctrines.

Convinced, as we profess ourselves to be, of the vast importance of the subjects treated of in this work, and admitting, as we freely do, the ability with which the author has treated them, it might be expected that we should enter into a detailed analysis of each chapter of the book; but such is not our intention. A great portion of a treatise on inflammation must, of course, be too purely elementary to claim a detailed notice or lengthened extracts. Having stated, therefore, our very favorable opinion of the work, we shall confine ourselves to giving a general sketch of its contents.

Mr. James, in the first place, considers the questions connected with the ordinary powers and state of circulation, the state of the vessels under inflammation, and the symptoms which manifest themselves in the part; secondly, he enters into the subject of sympathy, as connected with the extension of inflammation to the adjoining parts, and the influence reciprocally exerted between the system at large and the part inflamed: these are the materials of of the first chapter. In the second chapter, he examines the principal causes which either predispose to the production of inflammation in a part, or influence its progress when produced, whatever may be the original cause.

"The objects of inquiry may be divided into those more immediately depending upon the actual state of the system, as regards the condition of the nervous system; the digestive organs; the state of the circulating fluid, with reference either to its quality or quantity; the reciprocal influence of the solids and fluids, or circumstances connected with the condition of the part: and more remotely as regards the influence of temperament, diathesis, hereditary disposition, age, sex, habits, &c.; or extrinsic circumstances, as the influence of air, temperature, and climate." (P. 5.)

A separate consideration is the examination of the processes and products of inflammation, and their apparent purposes in the economy; and this forms the subject of the third chapter. In addition, although Mr. James confesses that the matter can never be completely handled apart from the consideration of the particular species of inflammation, he gives a *general* view of the objects of remedial means, the principles on which they are understood to act, and the circumstances which would guide us in adopting them; and thus concludes the first part of the work. The second part comprises an account of the particular nature and treatment of the various species of inflammation; the first division including those inflammations produced by external injuries; as mechanical and chemical injuries. The second, those arising from internal causes. Limited and spreading inflammations are considered in separate classes.

In the section "*on the State of the Vessels in Inflammation,*" the author controverts, with much ingenuity, the idea that this condition consists in mere debility of the capillaries; and he asks, as many have asked before, what is the difference between inflammation and blushing, or the distention of the erectile tissues?

We think Mr. James would have done well to notice, in this place, the recent experiments of Dr. Marshall Hall, given in his "Essay on the Circulation," chap. v. As these experiments throw a new ray of light on the question of the state of the vessels in inflammation, and as we happen to know Dr. Hall's opinion on this subject, both from having been witnesses of some of his experiments and from repeated conversations, we think it may not be out of place to give a short sketch of them here.

The first link in the chain of phenomena in inflammation is, according to Dr. Hall, not increased action nor mere debility, but an actual interruption to the flow of the blood through the minute and capillary vessels. This interruption may occur from causes acting *without* or *within* the vessels. The former induces such a change in the condition of the inner surface of the vessels as causes the globules of blood which move along them to *cohere* to it. Such globules immediately cease to move: if the vessel be a mere capillary, the circulation through it is immediately arrested; at an angle from which two capillary branches originate, a single globule is frequently seen to fix, whilst part of it is carried into each of the two branches, by which it acquires a crescent or kidney form; in a minute vein, globules are frequently seen cohering to the inner membrane, whilst others are pursuing their course in its more central part. All these facts prove that a change has been effected in the vascular lining. On the other hand, it is more than probable that many substances injected, or otherwise conveyed, into the circulating system, are carried along, until, arriving in the minute or capillary vessels, they are arrested in their course, and obliterate those vessels. In such cases, inflammation may be set up in various parts of the system simultaneously. This is actually seen, for instance, in phlebitis, in which the secreted pus is carried along the circulation to various parts, inducing in each inflammation, suppuration, destruction, &c. Dr. Hall purposes tracing these phenomena by means of the microscope: it is quite obvious, *a priori*, that they will be highly curious and interesting.

In the case in which the healthy globules of blood adhere to the lining membrane of the vessels, they are occasionally seen to be washed away one by one, and at other times, all at once, by the continued waves of fresh blood, brought in the course of the circulation. Is not this phenomenon the type of *resolution?*

At other times the adherent globules break down, their globular character being lost. Is not this the type and the rationale of the *formation of sanies and of pus?*

In a third instance, the texture of the part breaks up with that of the globules, affording the type of *destructive ulceration.*

An *abscess* is probably but the parts adjacent to globules first adherent, then broken down, and then forced asunder so as to leave a cavity. Such abscesses are solitary when the cause is an external one, or the seat of the inflammation simple and confined to the part: but when the cause is internal to the vascular system, when pus or other matters are carried along the circulation, they may be arrested in various parts, and give origin to abscesses in each, as are observed in phlebitis, &c.; or, with abscesses in one part, we may have destructive inflammation in another, as the eye, &c.

It is a singular confirmation of this view that a similar affection of the left eye has taken place in a case of the injection of a saline solution into the veins.* Dr. Carruthers states, in a report of such a case, on June the 24th, "her left eye, ever since the first injection, has been very much inflamed, and there is now" (the fourth day,) "a small ulceration on the cornea, a little below the centre."

It is no longer a difficulty to conceive the difference between real inflammation and blushing, or erythema. Nor is it more difficult to understand that the resolution of inflammation is a slow process, whilst that of erythema may be prompt; that the redness of inflammation disappears but partially, whilst that of erythema disappears totally under pressure.

But we must not go farther. We believe Dr. Hall is preparing a paper for a Medical Society on this subject, and on that of the formation of morbid structures, which we would not anticipate. We may add, that such investigations are really and truly the groundwork of the science of morbid anatomy, which must not longer be viewed as consisting of splendid pictures of morbid changes in their crude, brute masses.

Having, in the three first chapters, briefly stated those circumstances which commonly influence the production and progress of inflammation, Mr. James enters, in the fourth, "*on Remedial Means.*" The following observations on bleeding are worthy the attention of those who fancy that, provided they are armed with a lancet, they have nothing to fear from inflammation.

"As there is no circumstance which produces so direct an influence on most inflammations as the determination of blood to the

* See Medical Gazette for August 11, 1832, p. 607.

part, so there is no remedy of equal efficacy as bleeding; and we might almost persuade ourselves, while consulting the opinion of many eminent men on this point, that scarcely any other was required: such opinions must be admitted with great reserve; they are true only as regards some inflammations: we might also from the same sources almost be tempted to believe that it is hardly capable of doing harm, except it be carried exceedingly far. Now, although I most willingly concur in the opinion that it is the remedy for many inflammations, especially in the beginning, it should, likewise, at the same time, be stated that it is not by any means adapted for all inflammations, or for all stages of the same; that it ought not to be employed in many to a considerable extent, or that, in many others again, it is applicable at all.*

"A few examples will illustrate these points. We bleed in inflammation of joints, and in many of those of the eye, with almost a certainty that, if employed in time, and sufficiently, it will cure them, and with the confidence that we shall not endanger the lives of our patients; but we are not equally certain that we shall do the same good by this means in the advanced stage of these very inflammations.

"Again: we bleed, even to a greater extent, in inflammations of vital organs; and we do so in the beginning, because we know that, in the beginning, it will succeed, and we may find this the sole means to be depended upon for saving the patient's life; but we cannot always employ it, even in these cases, without great risk of harm, as well as great chance of good. We may accelerate the progress of effusion, often fatally; we may carry it so far as to prevent restorative actions, in which way many persons have died after injuries of the head from its abuse. We may adopt it in cases of inflammations of vital organs, in which it is unsuitable, as in thos which occur in the advanced stages of fever; in some kinds of uterine inflammation, &c.; and again, where its sparing employment may be advantageous, its free use may be fatal.

"Again: in inflammation of vital organs we find that its employment, or its extent, must depend mainly on the tissue inflamed: thus, as regards the lungs, the most ample depletion is called for in inflammation of their serous membranes; but it is otherwise when the mucous membrane is concerned.

"Again: in many cases of injury, such as lacerated or contused wounds, we may be obliged to bleed, and largely; but we bleed without any confidence that we shall surely serve our patient by it; for if we fail in subduing the inflammation, we only render him less capable of contending with the stages of suppuration or sphacelus which follow.

* "I may repeat here what I have stated elsewhere, that blood is not too largely determined to a part in inflammation, unless from a particular disposition; and our object is to remedy this disposition, which is not always best effected by bleeding."

"In many inflammations we need not bleed at all: it would be useless, if not injurious, in many cases of boils, common phlegmon, mumps, &c.

"In many cases we should do positive injury by it, although the inflammation is sudden and intense, as in cases of viper bites, &c., carbuncle, &c.

"In many other inflammations, it is a remedy most doubtful, yet most important, as in erysipelas, or diffuse cellular inflammation.

"In many cases it must prove fruitless, as in some where the inflammation is maintained by the presence of a foreign body which cannot be removed. In others, the state of the constitution renders it so; it relieves for a short time, but the symptoms infallibly recur. We might drain every drop from the body, and yet not cure the inflammation: this is the case in some inflammations about the heart, &c., and frequently in rheumatism.*

"If we regard these, and many other instances which might be cited, we should be disposed to think that the justly deserved commendations which this remedy has received ought to be much qualified; and that, when its advantages are set forth, the limitations should be strongly enforced. It may be right to add, that as there can be no doubt that repeated bleedings establish a state of the blood in which the inflammatory appearances manifest themselves, we may be commencing a habit which will, at a more remote period, reproduce the evil we now wish to avert: this is no objection, however, to its use when really required." (P. 162.)

Mr. James states it as his opinion that there is no appearance of the blood, *taken singly*, which will either warrant the employment or forbid the use of depletion. He considers the pulse a better criterion than the appearance of the blood. If a pulse too frequent is rendered less so, too full is reduced, too hard becomes soft, too small becomes freer, too slow or oppressed regains more its natural standard; rises under bleeding, as the term is: either of these changes marks a beneficial effect; but if the pulse becomes more hurried, easily compressible, soft, weak, vibratory, uncertain, without relief, further bleeding will rarely serve.

Mr. James very briefly considers the influence of the several medicines which have a direct anti-inflammatory tendency, as the tartrite of antimony, mercury, colchicum, bark. Considering the importance of the subject, we are inclined to think he is somewhat too concise in his remarks upon opium as a remedy in inflammation.

In his comments upon the treatment of erysipelas, Mr. J. adverts to the application of lunar caustic to the inflamed parts, as recommended particularly by Mr. Higginbotham. He believes that it succeeds oftener than any other application, but he would not be

* "When the digestive organs are very wrong, as in erysipelas, &c., that will prevent the curative effects of bleeding, however far we may carry it in many cases."

understood to say invariably; and, in cases where it does not prove beneficial, he thinks there is no reason to believe it injurious. He would seldom adopt the severe mode by incision, advised by Mr. Copland Hutchinson, Mr. Lawrence, and other surgeons, until the Arg. Nitr. had been tried and failed. But in the second stage of erysipelas, when suppuration or sphacelus may occur, it is then the duty of the surgeon, "beyond all question, to make incisions."

"Whatever difference of opinion may be entertained as to their propriety in the earlier stages, whatever reluctance we may then feel to put in force so painful a remedy, whatever the kind of erysipelas may be, it may now be stated that their employment is perfectly indispensable and imperative; for, as has been before said, when foul matter and sloughs form in any part, they immediately communicate an unfriendly disposition to all those surrounding, and the type of the constitutional affection changes, and when confined their influence is greatly increased. These circumstances not only obtain in the present case, but, as these matters are neither led to the surface by that process which is termed pointing, nor walled in by adhesions of the cellular membrane, not only is their residence in the parts particularly injurious, but their spontaneous discharge indefinitely delayed.

"Although there is often great difficulty in ascertaining the existence, or the situation, of such collections, yet an accurate examination will probably detect them. The spot may often be discovered by a sensation as if there were a quag under the skin, if that be pressed upon pretty firmly; for, although the skin itself remains hard and tense, the subjacent parts are not equally so; at these places too the colour is often more dusky, and if, in addition to this, there are phlyctenæ on the surface, there will be still greater reason for believing that matter is situated underneath: collections of pus, however, may form without any slough; and in these cases there is not the same dark appearance of the skin, nor of the phlyctenæ which exist when there is. In whatever species of acute inflammation this colour is observed, it commonly denotes the existence of, or imminent risk of, sphacelus under the skin; and sphacelus of the skin will commonly take place to give this exit, if an incision is not made.

"As the safety of the patient depends upon the timely evacuation of such matters, it is far better to err by making an incision unnecessarily, than by omitting to do so when it is required: from the former conduct no harm can accrue, from the latter the greatest. The plan I have long adopted has been that of thrusting into a part where there is reason to suspect such collections, a keen double-edged knife deeply, and, if pus follows, converting the puncture, which gives little pain, into an ample incision. It is necessary that this should be deep, as it has to penetrate the loaded skin, and large, as a free opening is required to give sufficient vent to sloughs and matter, and also to answer another very important purpose, to free the tension. If we make small openings, such will be the stress upon their edges, that they will rapidly ulcerate or slough, till by these processes they are rendered large enough: if we at once make them so, they will gape and immediately relieve the tension at the part, and presently of the whole limb, by giving vent to the deep-seated matters and allowing the serum to drain off. The bleeding also, even now, will be beneficial.

'Wherever the matter may be contained, then without hesitation openings should be made to discharge it, and they may be repeated as often as they are called for by the formation of fresh collections; for it is the property of this inflammation, after it has subsided in one part, to spread with the same mischievous consequences to others, and until all these are evacuated the disease will not end. I have seen instances when, after treatment has completely subdued the violence of the disease, and when there has been every reason to believe the patient free from danger, yet from foul collections, although of no great size, remaining unopened, lives have been lost; such collections as certainly would not endanger a person under any ordinary circumstances, but capable of producing extreme mischief to constitutions shattered as they have been by the effects of the disease; as a trivial misfortune will sink a ship which has been crippled by a storm.

"Some may doubt whether, in a state of the integuments bordering upon gangrene, incisions should be made: it is my own belief, that, whenever the affection of the cellular membrane predominates over that of the skin, they will be advantageous."

Having stated the general purposes of the work, and selected a few parts as specimens of the manner in which it is written, we must terminate our notice. It is at present the only systematic work on inflammation in the English language; and we should think that no other writer will now venture into the field in competition with Mr. James, who has so ably and so satisfactorily treated the subject.

Dr. Thomson's "Lectures on Inflammation," which possessed, as they deserved, a very high reputation, have for some time been out of print; and, even if they were still in the market, the preference must be given to Mr. James's work, as he brings the subject down to the improvements of the day, and has enriched this second edition by much instructive matter which he has derived, since he first published, from the labours of others, as well as from his own observation.

---

*Spasm of the Glottis in Infants.* By Dr. W. B. Joy. *Cyclopædia of Practical Medicine, Part IX., for September* 1832.

This article, by Dr. Joy, is upon a very important subject, of which we have yet much to learn, both as regards its pathology and treatment. We have paid much attention to the disease here denominated "spasm of the glottis," and have given a particular account of it in a work we published some years ago, on the Convulsions of Infants.* We have some objection to the term employed by Dr. Joy for the purpose of designating this disease, as it assumes a fact of which we yet require proof, namely, that in spasm of the glottis really consists the essence of the malady; and we should, therefore, still prefer the more indefinite, but yet correctly descriptive, title which we employed in the work referred to, "a spasmodic affection of the chest and larynx in young children, accompanied by general or partial convulsions." Dr. Joy correctly states, that this very formidable affection, which, as we well know from our own observation, is by no means of rare occurrence in infancy, was till late years but ill understood, being for the most part confounded with croup and other inflammatory affections of the air-passages. It might be supposed that MILLER and CHALMERS referred to this complaint under the name of the acute asthma of children; but, as these writers thought that, if early discovered, it might always be cured by assafœtida, and the subsequent employment of bark, we cannot bring ourselves to believe that the two diseases are the same.

"A further step towards the diagnosis of the disease was made by Underwood, in his division of croup into the chronic or spasmodic and the acute or inflammatory. The former of these, which seems to answer to what is now called spasm of the glottis, he states to have been known to continue so long as two months, and then to have yielded to opium. 'Instances have likewise been met with of children crouping for two or three days, and being then seized with hooping-cough, which has instantly removed the croup. These circumstances,' he continues, 'seem to prove that species of croup to be truly spasmodic. I have seen it frequently in this form attend the cutting of teeth, being then the mere consequence of irritation, as we see cough and various other symptomatic affections induced at this period.' In addition to assafœtida and bark, Underwood recommends the occasional use of emetics and cicuta, 'one or other of which must be persevered in as long as any symptom of the disease, and particularly the croaking noise, shall remain.' He seems, however, to have had but a very imperfect notion of the disorder, confounding it in one part of his work with croup, with which it has really no affinity, the membrane lining the larynx presenting no traces of diseases on dissection; and treating of it in another place under the head of inward fits, which is an imaginary disease. Dr. Ferriar, likewise, in recognizing a spurious species of croup, seems to have met with the affection in question, though he did not attain to a correct idea of its nature.

"One of the first accurate accounts of the disease is that by Dr. John Clarke, in his Commentaries on the Diseases of Children. He describes it under the name of 'a peculiar species of convul-

* Practical Observations on the Convulsions of Infants. By JOHN NORTH. 1826.

sion in infant children," often miscalled spasmodic or chronic croup. The child, according to this writer, is suddenly seized with spasmodic inspiration, consisting of distinct attempts to fill the chest, between each of which a squeaking noise is often made; the eyes are staring, and the child is evidently in great distress. The face and extremites, if the paroxysm continue long, become purple; the head is thrown back and the spine bent, as in opisthotonos; at length a strong inspiration takes place, a fit of crying generally succeeds, and the patient, much exhausted, falls asleep. The paroxysm may occur often in the course of the day, and is most apt to take place on awaking, or on exposure to slight causes of irritation. If neglected, it may go on recurring frequently for two or three months, until at length, general convulsions ensuing, the parents become alarmed. It seldom occurs, he thinks, after the third year, or in children who have lived by suckling alone till they have got some of their teeth, and thus escaped those derangements of the health which are connected with an unsuitable diet in extreme infancy. This, as well as other convulsive affections of children, he ascribes to disease of the brain, which may be induced, he thinks, by overfeeding, keeping the head too hot, the sudden cure of ophthalmia or of cutaneous eruptions, the occurrence of fevers, &c. In one fatal case he discovered, on dissection, both fulness in the vessels of the brain and water in the ventricles.

"A peculiar species of hydrocephalus, ushered in by spasm of the glottis, has been mentioned by Monro; and a case which seems to have been of this kind is also alluded to by Underwood. Gölis, in treating of the predisposing causes of hydrocephalus, places this affection amongst them, and describes it as 'a peculiar disorder of respiration, in which infants, after sudden waking out of sleep, or from terror or anger, often too without any cause, are suddenly seized with a deep shrill respiration, which for many seconds, sometimes even for minutes, threatens suffocation; the whole body becomes stiff; the face, hands, feet, and particularly the finger and toe nails, black or blue, and the little patients lose their breath and consciousness; at length, however, with a cry of alarm they again recover both."

"Dr. Cheyne has given, in the following passage from his work on Hydrocephalus, a more satisfactory account of this affection, since called spasm of the glottis, than any of his predecessors: 'Another disease of infancy requires to be briefly adverted to in treating of the diagnosis of hydrocephalus. This disease has been known to some authors under the titles of inward fits, chronic croup, &c. It begins with a crowing inspiration, like that which takes place in the commencement of a paroxysm of pertussis. As at first there are long intervals between these spasmodic inspirations, (several days perhaps;) as they appear to be connected with a disordered stomach and absence of bile in the bowels; to arise from sudden exertion, or fits of passion; and as the child often continues to thrive notwithstanding, the disease is not much attended to. At last, however, the spasmodic inspirations excite just alarm; they occur frequently without any apparent cause, when the child is perfectly tranquil; the complexion becomes purple, insensibility follows, and not unfrequently universal convulsions or rigidity of the muscles, with the thumbs clenched in the hands: these convulsions, in seven instances to my knowledge, have ended in death. However, after continuing many weeks, or even months, this affection often terminates favorably with the cutting one or more of the teeth, or it may be relieved by effectually scarifying the gums, changing the air and diet, and alternating mercurials with carminative purgatives. The pathognomonic of this disease is a crowing inspiration with purple complexion, not followed by cough. In some cases this affection is attended not merely with a permanent clenching of the hand upon the thumb, but also with a very remarkable fixed spasm of the toes, particularly the great toe, which gives a look of swelled deformity to the upper part of the foot." (P. 349.)

In the twelfth volume of the Edinburgh Medical Journal, Dr. Kellie has given a very minute description of this particular "swelling of the tops of the hands and feet," which had been mentioned by Dr. Underwood as a symptom of painful dentition.

"Spasm of the glottis cannot be considered a rare disease, as Cheyne had seen previous to the year 1819, when his work was published, no less than twenty cases, of which one third were fatal. He, like Clarke, has no doubt that the brain is the seat of the disease; but what is the precise morbid condition of this organ giving rise to these peculiar symptoms, or whether there be any invariable one, has not yet been accurately made out. The results of dissection in three cases are given in the appendix to his work. In the first of these, two tumors, apparently of a scrofulous nature, were found imbedded in the substance of the brain; in the second, the convolutions were nearly obliterated, and the cerebral substance appeared uncommonly firm; in the third, the veins on the surface of the brain were turgid with blood; a considerable quantity of serous fluid existed between the tunica arachnoides and the pia mater, giving a gelatinous appearance to the surface of the hemispheres; whilst about an ounce of water was discovered in the ventricles. Disease of the brain has not, however, always been detected. Thus, in two cases mentioned by Dr. Merriman, of children who died in these fits, no appearance of cerebral affection could be discovered. The principal deranged structure was a collection of small glandular swellings in the neck pressing upon the par vagum. In none of these cases was there any trace of inflammation in the larynx or trachea." (P. 350.)

Dr. Merriman also has mentioned this disease of infants: he thinks it by no means an uncommon affection of children; and attributes it chiefly to improper food and close and confined apartments. We do not doubt that either of these causes would aggravate the complaint, but, in the many obstinate and tedious instances we have seen of it, the greatest attention was paid to the diet of the child, and the apartment in which the little patient lived was neither close nor confined.

Dr. Joy informs us that Wichmann, the Hanoverian physician, and Schmalz, in his work on Diagnosis, have taken particular pains to point out the distinctions which exist between this disease and croup. This trouble is somewhat unnecessary, as we think it scarcely probable that any practitioner, with even moderate attention and experience, could confound them.

"Spasm of the glottis has been described by Mr. Pretty in the Medical and Physical Journal under the singular name of cerebral croup. It has also been noticed by Richter, Henke, Jahn, and several other writers in Germany; and in France it has been treated of by Guersent under the title of 'pseudo-croup nerveux.' Though it has been thus recognized by so many respectable authorities as a distinct and well characterized disease, yet Autenreith and Hecker, and more recently Jurine and Albers, have treated it as a mere modification of croup. From this, however, we repeat, it is broadly distinguished, as well by its intermittent nature as by the usual absence of cough and fever, and by the sudden death which not unfrequently occurs in it. Even if a slight cough happens to co-exist, the disease may still, as Dr. Hamilton has remarked, be satisfactorily distinguished by the cough not being a peculiarly hoarse one, and by the breathing in the intervals remaining perfectly free.

"The latest, and by much the best account of the disease, is that by Dr. Marsh. The disorder, as described by this writer, begins by the muscles of the glottis; but if neglected or mismanaged, it may extend to those of the extremities, and even terminate in universal convulsions. Its occurrence is by no means rare, and its result not unfrequently fatal. It sometimes appears to be a purely idiopathic affection; but in far the greater number of cases, as Dr. Marsh observes, 'it is complicated with painful dentition, derangement of the digestive functions, a cachectic state of the system induced by an impure atmosphere, fever, and occasionally with effusion into the ventricles of the brain. The child is observed to awake suddenly from sleep in a state of alarm and agitation, to struggle for breath, and after repeated efforts, to recover from the paroxysm with a long and sonorous inspiration,' usually described by nurses as a whoop or crowing sound. The face is swollen and purplish during the fit. As the disease advances, similar attacks occur even while the child is awake, 'sometimes without any perceptible cause, but more frequently when it is vexed and about to cry.' Robust as well as delicate children are liable to it, but it especially attacks those which are of a passionate and irritable disposition, and the subjects of it are observed to be easily startled even by the slightest noises. It is only in its more advanced stage that Dr. Marsh has observed the peculiar swollen state of the hands and feet, and the rigid contraction of the thumbs and toes already alluded to." (P. 350.)

In the treatment of the disease, the chief indications consist, says Dr. Joy, in removing any complications which may exist, and in improving the general health, and especially the tone of the nervous system. Difficult dentition he regards as a very frequent exciting cause of the affection; and when it is suspected to have this origin, the swollen gums must be immediately divided. In those cases where a careful investigation into the state of all the functions of the body detects no complication, he recommends a mildly tonic plan of treatment. "The sulphate of quinine, or some of the mineral tonics, are here found very useful."

"Antispasmodics too often disappoint expectation, and are of very secondary importance in the treatment of the disease: ether and ammonia are the only medicines of this class in which Dr. Clarke had the slightest confidence. The Tinctura Fuliginis of the older Pharmacopœias is spoken of by Dr. Marsh, with some commendation. In all cases we should endeavour to improve the general health and strengthen the nervous system, by country air, a well-regulated diet, and attention to the state of the bowels. By giving the child the advantage of a pure air, and of a succession of good nurses, during the whole period of dentition, more good has appeared to be done than by all other measures put together. Dr. Marsh remarks, that all his cases occurred in children of a scrofulous constitution; a fact which leads him to insist still more strongly on the importance of a pure atmosphere, healthy nutriment, and tonics. Dr. Cheyne, also, as we have seen, dwells much on the utility of change of air and of diet. Free exposure to the open air, and daily sponging the body with cold water, are amongst the most effectual means we possess for lowering the nervous excitability in this and many other spasmodic disorders.

"When the convulsions threaten to become general, leeching the temples, cold applications to the head, and fomentations of the extremities, will generally be required. In the case of a delicate child, of two years old, mentioned by Marsh, in which the paroxysms were of very frequent occurrence, and were accompanied by general convulsions, a tobacco enema (five grains infused in six ounces of water,) was administered: it produced its specific effect in a very marked manner, and no appearance of convulsions were observed for a month after. Removal to the country appeared to confirm the cure. The symptoms, however, recurred on again returning to town, to a house which had been recently painted. Several other instances are mentioned, where the disease seems to have been excited by the unhealthy atmosphere of newly-painted rooms.

" Dr. Merriman is of opinion that this disease, when early attended to, will commonly yield to aperients, so given as to procure at least two copious evacuations daily, together with the continued use of soda, or a strong infusion of burnt sponge, and proper attention to the diet and regimen: 'When the head is manifestly affected, cupping glasses behind the ears are required; but when the patient has cold, pale, and flabby cheeks, abstraction of blood is rather injurious than beneficial.' Dr. Hamilton has found Dalby's carminative a useful medicine in this affection, after the bowels have first been freely opened. We have known the disorder, after resisting the influence of purgatives and change of air, cease finally on the occurrence of a spontaneous diarrhœa.

"Spasm of the glottis has occasionally been mistaken for an inflammatory affection of the lungs or air-passages, and been much exasperated by a consequent perseverance in the antiphlogistic treatment, and by confinement to the close air of a heated apartment. Even in its simplest and mildest form, it should never be neglected, as, in the absence of every complication, the spasm of the muscles of the larynx alone has often proved suddenly fatal. Dr. Johnson knew a case recovered by the immediate employment of artificial respiration, after death had apparently taken place.

"Before terminating this article, we may state that fatal spasm of the glottis in adults occasionally takes place, under the influence of irritating causes in the neighbourhood of the larynx. Thus, in an interesting case recorded by Mr. Kirby, spasm of the glottis and death were caused by the irritation of a mouthful of food sticking in the œsophagus; and we have seen a patient with many of the symptoms of acute laryngitis die rather unexpectedly, when, on dissection, very little, if any, redness or swelling were discovered in the membrane lining the larynx, and scarcely any other morbid appearance than one or two minute ulcers. It is probable that the fatal result, as well as the sonorous breathing, and many of the other symptoms in such cases, depend on a spasmodic contraction of the glottis." (P. 351.)

There is one very important fact, which is properly noticed by Dr. Joy. It not unfrequently happens that children are very suddenly destroyed by this spasmodic disease, and we would, therefore, caution every practitioner who has a case under his care to give a very guarded prognosis, however mild the symptoms may appear to be, and however completely the child may rally in the intervals between the attacks. We are the more anxious to state this fact, as, when we first wrote upon the disease, we stated that we believed "such an occurrence to be very rare, considering the very great frequency of the complaint." Subsequent experience, however, has induced us to change our opinion. In the course of a few weeks, four cases occurred in our own practice, in all of which death suddenly took place during a violent attack of general convulsions and difficult breathing. In neither of these instances was a post-mortem examination permitted.

As we have had frequent opportunities of watching the commencement and progress of this disease in young children, we may be permitted to give a short account of it, as it has been presented to our own observation. For a more detailed description, we refer to the work above mentioned. The premonitory symptoms occur at an uncertain age, but generally between the third and seventh month. At first they may not be sufficiently striking to attract the particular attention of the friends, although the practitioner, who has met with similar cases, will with confidence predict from them the symptoms which are subsequently to be developed. When the child wakes from its sleep, the breathing is for some moments unusually accelerated, and is accompanied by that kind of noise which an increased secretion of mucus in the air-passages would produce. The countenance of the child soon becomes anxious; the sides of the nose are drawn in; the face becomes pallid and emaciated; the upper lip appears swoln, and is sometimes of a purplish cast; the child frowns almost constantly. When put to the breast, the child sucks greedily for a moment, but suddenly ceases to do so, throwing back the head with violence. The bowels are usually obstinately constipated. A considerable time may elapse before any remarkable change takes place in these symptoms. A spasmodic affection of the hand is usually the next symptom which excites attention. The thumb will now be found constantly and firmly pressed upon the palm of the hand; the wrist and ankle-joints are bent rigidly inwards; the head is almost constantly thrown backwards, by which the anterior muscles of the neck are kept painfully on the stretch. The inconvenience at the moment of waking is no longer a mere acceleration of the breathing: this symptom is now aggravated, but the noise accompanying respiration has gradually assumed a very different character from that which at first marked it. Each inspiration is now attended by a loud crouping noise, which may be heard in an adjoining apartment. The chest and larynx appear painfully constricted; the heart palpitates violently; the child sobs, but never cries in its natural manner during these paroxysms of suffering. So great is the difficulty of breathing, that it sometimes appears to be almost totally suspended for a few seconds. The child has frequent attacks of convulsions, the paroxysms of which vary in duration and violence. In two cases we have seen, both of which terminated favorably, the state of opisthotonos was so complete that for many days the head and heels were the only parts which touched the bed. In the majority of cases there are no symptoms of fever, nor any indication of determination of blood to the head. In three instances, however, we have seen febrile symptoms superadded to those above described, together with evident cerebral derangement and determination of blood to the brain. The firm contraction of the thumb, and the rigidly bent position of the hands and feet, and the croupy sound during respiration, often continue for many weeks without intermission. The child sometimes appears lively for a short period, and the countenance may be animated by a momentary gleam of cheerfulness, but it almost invariably awakens from its unsound slumbers with a convulsive paroxysm.

The proximate cause of this affection has not yet been determined. In many cases it is certainly connected with painful dentition. We have known all the symptoms gradually disappear upon the appearance of teeth, and instant relief will be frequently afforded if dentition has sufficiently advanced to admit a free division of the gums.

Much discrepancy of opinion exists as to the degree in which the brain is affected in this complaint. It is the opinion of some practitioners of celebrity, that, " if a child makes this crouping noise in breathing," that hydrocephalus will inevitably ensue, unless large doses of calomel and bleeding are had recourse to. It has already been stated, that, in connexion with the above symptoms, we may have sufficient evidence of cerebral derangement to justify a vigorous and prompt mode of treatment: but, in the great majority of instances, we have no proof of affection of the head: nor have we any right to assume that certain individual symptoms, such as the crouping noise, or bent thumb, must necessarily be followed by serious affections of the brain; and the proof of this assertion is afforded by the fact of the frequent subsidence of the symptoms, without injury to the patient. Capuron has described this disease with some accuracy, and he observes that, upon dissection, no trace of inflammation is to be discovered, and that there is no collection of mucus in the air-passages or in the lungs. In our own practice we have never seen the state to which Dr. James Johnson applies the term of " carpo-pedal spasm," unconnected with symptoms of painful spasmodic action of the chest and larynx.

The treatment of this complaint must be conducted upon general principles. Mild purgatives are almost always necessary. The propriety of taking away blood must be determined by the symptoms and constitution of the child; but we believe depletion is rarely required. If the gums are swoln and expanded upon the surface, they should be freely divided. If the gum is not perfectly divided down to the tooth, no benefit can be derived from the operation. We may take this opportunity of observing that the principle upon which this simple operation is performed is not generally understood. Relief is afforded by it, not merely by the division of the gum, but by the incision being made down to the tooth, in consequence of which it rises a little, and no longer presses upon the highly sensible and vascular pulp beneath the root. One free incision of the gums may be sufficient for the incisores and canines; but a crucial incision is always necessary when it is our object to liberate the molares. We are not unfrequently urged by the parents of the child to give sedative medicines to procure tranquil sleep, and to relieve the spasmodic and difficult breathing which forms the principal and most distressing part of the complaint; but we believe that the internal use of such remedies is rarely beneficial. If sedatives are given, we should prefer small doses of extract of conium or hyoscyamus. Rubbing the chest and spine, briskly and frequently, with a liniment composed of equal parts of laudanum, spirits of camphor, and soap liniment, is often very serviceable. Blisters are not only useless, but prejudicial, on account of the irritation they produce. In more than one instance we have known the attack return with all its original severity, in consequence of the child being suddenly awakened, either by some accidental noise or by the imprudence of the nurse; and, as there appears to be a greater tendency to the occurrence of the most severe symptoms when the child rises from sleep, any excitement, even of the most trifling kind, should at that time be carefully avoided.

